# Brain Activity in Visual-Motor Illusions With Enhanced Joint Motion Intensity

**DOI:** 10.7759/cureus.65786

**Published:** 2024-07-30

**Authors:** Junpei Tanabe, Kazu Amimoto, Katsuya Sakai

**Affiliations:** 1 Department of Physical Therapy, Hiroshima Cosmopolitan University, Hiroshima, JPN; 2 Department of Physical Therapy, Faculty of Rehabilitation, Sendai Seiyo Gakuin College, Sendai, JPN; 3 Graduate School of Human Health Sciences, Tokyo Metropolitan University, Tokyo, JPN

**Keywords:** functional near-infrared spectroscopy, kinesthetic illusion, brain activity, maximum force, visual-motor illusion

## Abstract

Background

Visual-motor illusion (VMI) is a cognitive approach used to evoke kinesthetic sensations. Research suggests that VMI can modulate brain activity depending on the specific joint movement observed. This study aimed to identify differences in brain activity when observing video images of joint movements at different intensities of movement in VMI.

Methodology

The study included 14 healthy adult participants. Two types of video images were used: pure ankle dorsiflexion movements (Standard-VMI) and ankle dorsiflexion movements with added resistance (Power-VMI). The brain activity measurement protocol employed a block design with one set of 15 seconds rest, 30 seconds VMI task, and 30 seconds follow-up. Each participant performed the VMI task twice, alternating between Standard-VMI and Power-VMI. Brain activity was measured using functional near-infrared spectroscopy, focusing on motor-related regions. Subjective impressions were assessed using visual analog scales (VAS) for kinesthetic illusions.

Results

The results revealed that Power-VMI stimulated significantly greater brain activity in the premotor and supplementary motor cortex, supramarginal gyrus, and superior parietal lobule compared with Standard-VMI. Power-VMI resulted in higher VAS values for kinesthetic illusion than Standard-VMI. Additionally, a positive correlation was observed between brain activity in the superior parietal lobule and the degree of kinesthetic illusion.

Conclusions

These findings indicate that Power-VMI enhances both motor-related brain areas and motor-sensory illusions, potentially having a greater impact on improving motor function. This study provides valuable insights for developing VMI interventions for rehabilitation, particularly for individuals with paralysis or movement impairments.

## Introduction

Visual stimuli can be used to induce kinesthetic sensations and have been applied in rehabilitation [1]. Recently, visual-motor illusion (VMI), in which visual stimuli presented in a self-motion image induce kinesthetic illusion, has been reported [2]. VMI has been shown to be an effective cognitive approach for paralyzed upper and lower limbs in hemiplegic patients through visual stimulation [2,3]. VMI involves recording the movement of the non-paralyzed limb, superimposing this image onto the participant’s actual immobile limb, and creating an illusion of movement where none exists [2,3]. A similar intervention method, mirror therapy (MT), produces illusions from visual stimuli and may promote interhemispheric inhibition because it requires movement of the nonparalytic side [1,2]. VMI does not require movement of the nonparalytic side and thus can passively induce the illusion, allowing subjects to focus on the visual stimulus.

There have been several neurophysiological studies on VMI. For instance, Kaneko et al. used functional magnetic resonance imaging (fMRI) to measure brain activity during VMI for finger movements in healthy participants [4]. The results showed that VMI elicited a sense of ownership (SoO) in the monitored hand and activity in the frontal-parietal network (premotor cortex, supplementary motor area, and inferior parietal lobule). This activity, similar to that during actual movement, is associated with the region of the brain involved with motor imagery [4]. Furthermore, using functional near-infrared spectroscopy (fNIRS), Wakata and Morioka reported that VMI induces a sense of agency (SoA) and increases prefrontal cortex activity [5]. Sakai et al. used fNIRS to measure brain activity during VMI on the ankle joint and reported increased activity in the premotor cortex [6]. Based on this, VMI not only increases brain activity in motor-associated regions but also induces motor imagery and embodied sensations (SoO and SoA).

However, an assessment of the methodologies of previous VMI studies revealed that the videos used depicted simple movements such as hand flexion-extension, wrist palmar flexion-dorsiflexion, and ankle plantar dorsiflexion (Standard-VMI (S-VMI)) [2-6]. In a previous study, brain activity was measured by fNIRS during simple and complex finger movement MT in stroke patients, with more ipsilateral M1 activation in the complex task [7]. In motor imagery studies, complex movements also increased activity in the premotor cortex, posterior parietal, and cerebellar regions compared to simple movements [8]. These brain regions are strongly associated with cognitive aspects of motor control and motor processes, such as motor selection, preparation, and motor imagery [9]. Thus, brain activity changes depending on the content of the visual stimulus and the task being imagined.

VMI may alter brain activity depending on the joint movements shown in the video [4]. Therefore, brain activity may also change in VMI due to differences in the visual stimuli presented. Ishizaka et al. reported that when healthy participants performed maximum-intensity toe flexion exercises, the activity in the primary sensory-motor cortex, premotor cortex, and supplementary motor cortex was greater compared with those observed in moderate-intensity exercises [10]. Therefore, increasing the intensity of joint motion in the video presented in VMI may increase brain activity in motion-related regions.

Based on these hypotheses, we previously performed Power-VMI (P-VMI), which adds resistance to the ankle joint to increase the strength of joint motion, and S-VMI in the paralyzed ankle joint of hemiplegic patients, comparing their effects on the sit-to-stand ability. The results showed that P-VMI improved the function of the paralyzed ankle joint and shortened the sit-to-stand time compared to S-VMI [11]. However, we have not examined the brain activity at different joint motion intensities in the presented images, and no such activity has been reported in previous studies. If there are differences in activation of motor-related regions depending on the joint motion intensity of the presented video, this could be an important finding for more effective VMI. Therefore, this study aimed to compare brain activity induced by S-VMI and P-VMI and examine changes in brain activity due to different joint movement intensities presented to the participants.

## Materials and methods

Participants

The effect size of the previous study reporting brain activity during VMI on the ankle joint in healthy subjects was 1.05 [6]. Based on these results, the sample size was calculated with a power of 0.95 (α error = 0.05) and was obtained according to the effect size of previous studies [6] using G*Power 3.1.9.2. Thus, the sample size was at least 12 participants in total. Inclusion criteria were: 1) right-handedness; 2) no history of orthopedic disease; and 3) no history of neurological disease [4]. Exclusion criteria were those who did not report the illusion in pre-experimental practice [4]. The study included 14 right-handed healthy adults (mean age 26.1 ± 3.5 years, 7 males and 7 females, Chapman’s dominant foot test: 12.6 ± 2.2 points). The study’s aim was explained to the participants, and written informed consent was obtained. The study was conducted with the approval of the Ethics Committee of Tokyo Metropolitan University (approval number: 19093) and conforms to the ethical standards established in the 1964 Declaration of Helsinki.

Types of VMI

**Figure 1:**
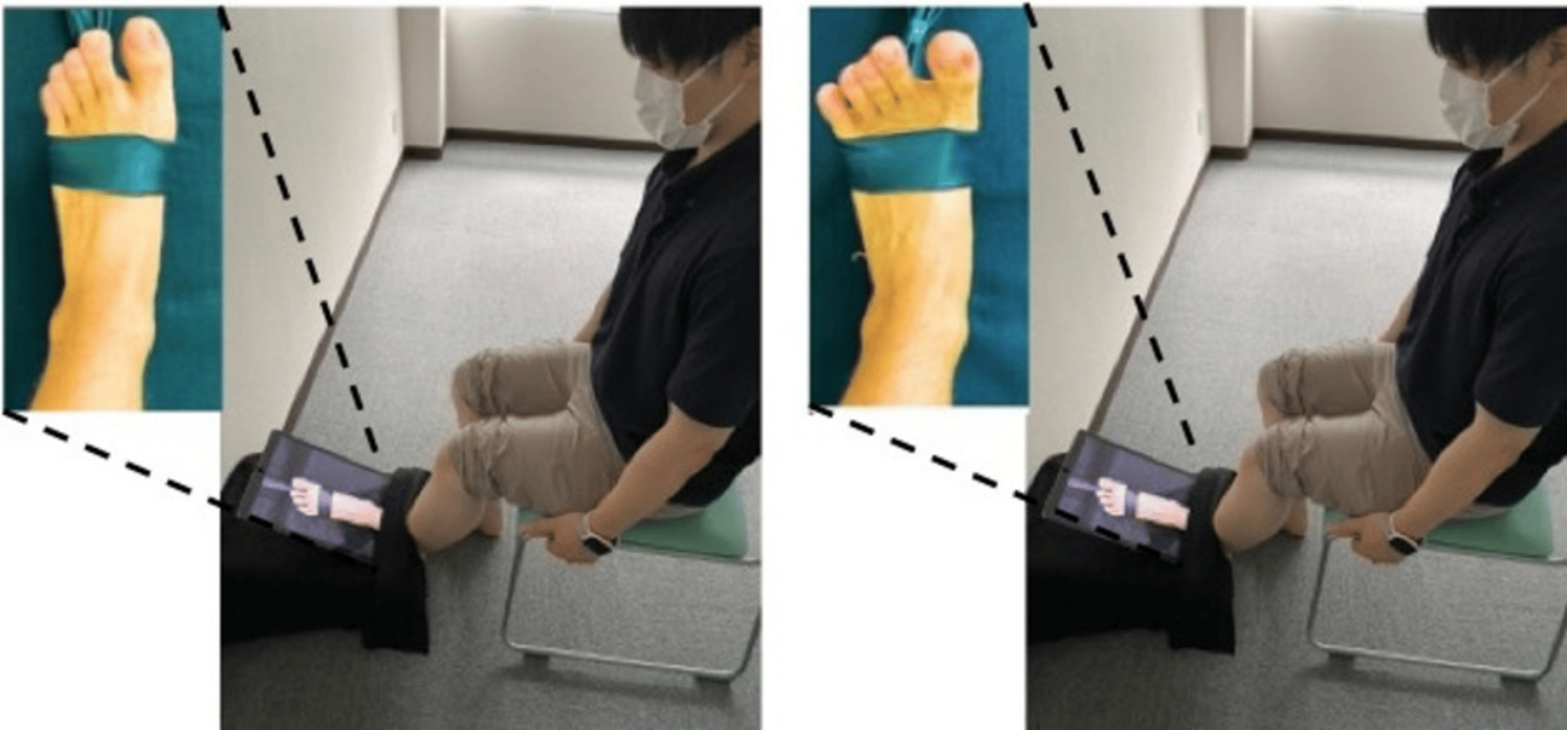
S-VMI and P-VMI. Standard visual-motor illusion (S-VMI) (left): Participants observed images of the ankle joint with Thera-bands wrapped around, without any resistance applied. Power visual-motor illusion (P-VMI) (right): Participants observed a video in which a Thera-band was wrapped around the ankle joint and resistance was applied. Compared to S-VMI, P-VMI revealed strong contraction of the tibialis anterior muscle, extension of the toes, and activity of the extensor hallucis longus muscle tendon and extensor digitorum longus muscle tendon.

S-VMI consisted of an ankle dorsiflexion performed using a Thera-band (Thera-band, Abilities, Tokyo, Japan) wrapped around the foot with no tension applied (Figure 1). P-VMI was a video image displaying dorsiflexion using maximum force, with resistance applied to the ankle joint by the Thera-band (Figure 1) [11]. Both images featured the Thera-band, but one image showed the band without tension (S-VMI), while the other illustrated it with resistance (P-VMI).

Experimental procedure

Before the experiment, videos for the VMI were recorded, capturing ankle joint movements of the dominant foot. Both VMIs presented 60 ankle dorsiflexion movements per minute, and the video was recorded on a tablet PC (iPad Pro, Apple, Cupertino, California). The video was then inverted using video inversion software to simulate left ankle joint (non-dominant foot) dorsiflexion exercises. The tablet PC was set to display the left ankle joint dorsiflexion motion. Under each experimental condition, participants sat in a chair, maintaining a resting position. Before the experiment, participants performed P-VMI and S-VMI for 30 seconds each as practice and answered whether the images being presented were P-VMI or S-VMI. During the practice, visual monitoring was constantly conducted to ensure that the participant’s left leg was not moving [4]. No participant’s left leg moved during the practice. Then, during the VMI task, the video on the tablet PC was continuously projected onto the left ankle joint (Figure 1). Participants were instructed as follows: “You do not actually have to move while watching the video; just imagine yourself moving” [11].

Design of brain activity measurement

**Figure 2:**
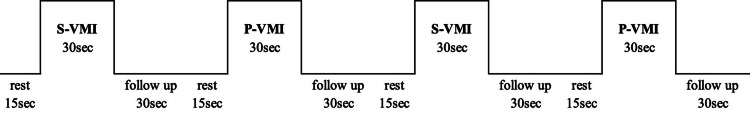
Measurement protocol. The tasks were performed in the order of S-VMI to P-VMI, two sets each. Brain activity was measured for a total of 300 seconds. The participant was instructed to look at the front marker during rest and follow-up. S-VMI: standard visual-motor illusion, P-VMI: power visual-motor illusion.

The brain activity measurement protocol employed a block design, with one set comprising 15 seconds of rest, 30 seconds of task, and 30 seconds of follow-up. This measurement protocol is presented in Figure 2.

Evaluation of sense of embodiment and kinesthetic illusion

The evaluation of the sense of embodiment (SoO and SoA) and kinesthetic illusions that occurred during the VMI intervention was based on the perceptions of the participants, assessed using a visual analog scale (VAS: 0 mm (no sense of embodiment or kinesthetic illusion) to 100 mm (sense of embodiment or kinesthetic illusion is occurring)) [4]. For kinesthetic illusion, participants were asked, “How much did your ankles feel as if they were moving?” Regarding SoO, participants were asked, “How strongly did the ankle joint in the video feel like part of your own body?” For SoA, participants were asked, “How much control did you feel you had over the movement of your ankle joints?” The sense of embodiment and kinesthetic illusions were assessed after measuring brain activity.

Evaluation of brain activity

**Figure 3:**
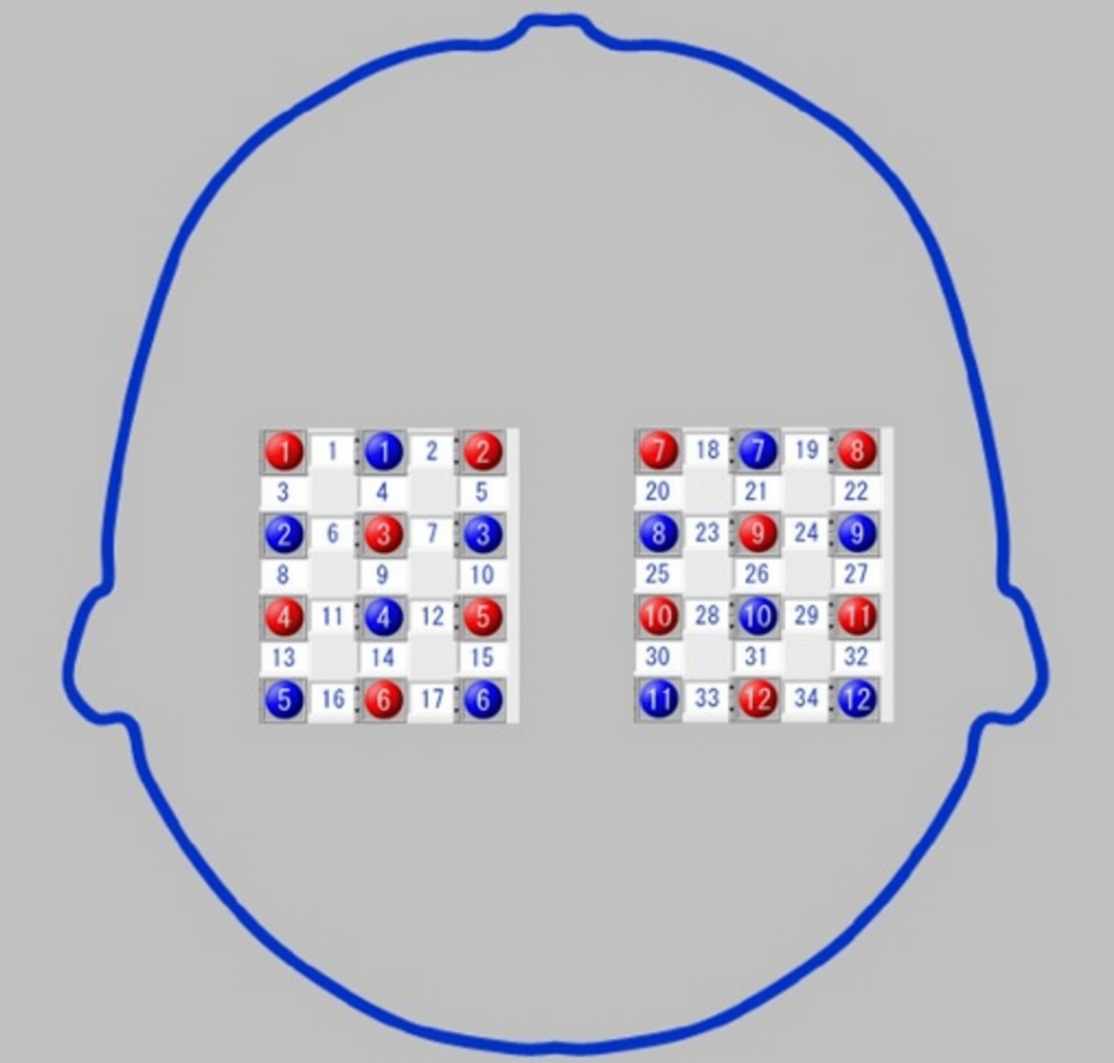
Channels and the region of interest (ROI) locations using functional near-infrared spectroscopy (fNIRS). Channels and ROIs were set with reference to previous studies [14]. The probe arrangements consisted of 34 channels, using 24 probes (12 sources and 12 detectors), set in a grid of 3 × 4 columns. Each probe was spaced 30 mm apart. The ROIs, as identified by near-infrared spectroscopy–statistical parametric mapping using a three-dimensional digitizer, included the dorsolateral prefrontal cortex (channels 1 and 19), frontal eye fields (channels 2 and 18), premotor and supplementary motor cortices (channels 3–7 and 20–23), primary somatosensory cortex (channels 8, 27, and 28), primary motor cortex (channels 9, 10, 12, and 24–26), supramarginal gyrus (channels 11, 13, 14, 29, 31, 32, and 34), superior parietal lobule (channels 15, 17, 30, and 33), and the angular gyrus (channel 16).

Brain activity was measured using fNIRS (LABNIRS, Shimadzu Corporation, Kyoto, Japan). The fNIRS is based on the modified Beer-Lambert method [12] and was used to measure the levels of oxygenated and deoxygenated hemoglobin (oxy-Hb and deoxy-Hb, respectively). The oxy-Hb signal, being more sensitive than the deoxy-Hb signal, was primarily analyzed as an indicator of brain activity [13]. The fNIRS system used was a continuous wave type, warmed up for at least 30 minutes before recording. The system wavelengths were 780, 805, and 850 nm [14]. Probe positions were 3 x 4 left and right, establishing a total of 24 probes (12 sources and 12 detectors) for 34 channels (Ch) (Figure 3) [14]. Previous studies have shown that VMI activates motor-related regions such as the frontal-parietal network [4], so the probe location was primarily in the frontal and parietal lobes. The probe distance was set at 30 mm with a sampling rate of 50 Hz. The midpoint of the probe, placed according to the International 10-20 method at the nasal root, occipital tubercle, and right and left external auditory canals, served as the Cz of the whole-head holder. The probe position was chosen to cover the parietal area. The regions of interest (ROIs) in fNIRS channels and brain locations are shown in Figure 3.

fNIRS analysis

Oxy-Hb data with low signal-to-noise ratios in multiple channels due to inadequate light sources, improper placement of detectors [13], and data containing visually clear motion artifacts from fNIRS were eliminated. For Oxy-Hb, a 0.01-0.1 Hz bandpass filter was applied to the fNIRS signal [15,16]. This filter effectively removed the effects of physiological activities such as Mayer waves, respiration, and heartbeat [15]. All channels were marked with 10 to 20 system landmarks (nasion, inion, right, left, and preauricular points) and recorded using a three-dimensional (3D) digitizer (3 SPACE®, FASTRAK®, Polhemus Co., Colchester, Vermont) to identify the brain regions corresponding to each channel position. All channels were then transformed using near-infrared spectroscopy-statistical parametric mapping (NIRS-SPM), and these coordinates were converted to 34 channel positions based on the estimated Montreal Neurological Institute (MNI) space [17]. NIRS-SPM was utilized to generate 3D functional images into MNI space using digitized data and probabilistic registration referenced to landmark positions by the 10-20 system [15]. The ROIs are shown in Figure 3.

Statistical analysis

Oxy-Hb was calculated as the sum average of rest, task, and follow-up values, respectively, and the amount of change was calculated as the Online effect (task value - rest value) and After effect (follow-up value - rest value). The Shapiro-Wilk test was used to confirm the normality of the Online effect and After-effect values for each Ch. For each measurement, Ch, S-VMI, and P-VMI were compared for each Online effect and After-effect using the Wilcoxon signed-rank test. The effect size, r, was calculated by dividing the z-score from each trial by the square root of the sample size. Effect sizes were interpreted as small (>0.1), medium (>0.3), and large (>0.5) based on Cohen’s guidelines [18]. The degrees of sense of embodiment and kinesthetic illusion in S-VMI and P-VMI were also compared by a paired t-test after confirming normality with the Shapiro-Wilk test. Furthermore, the relationship between tasks with significantly higher amounts of change and the degree of sense of embodiment and motor illusion was analyzed using Spearman’s rank correlation coefficient. IBM SPSS Statistics for Windows, Version 20 (Released 2011; IBM Corp., Armonk, New York) was used for all statistical analyses. The statistical significance level was set at p < 0.05 for all tests.

## Results

Comparison of brain activity for the task

All participants successfully determined P-VMI and S-VMI. The results of the comparison of brain activity for the task are detailed in Table 1. For the Online effect, P-VMI demonstrated a significant rise in Oxy-Hb over S-VMI in the left premotor and supplementary motor cortex (Ch 6) (Z = 2.272, n = 14, p = 0.023, ES r = 0.61). For the After-effect, P-VMI displayed significantly greater increases in Oxy-Hb than S-VMI in the left premotor and supplementary motor cortex (Ch 6), left supramarginal gyrus (Ch 13), and right superior parietal lobule (Ch 30) (Z = 2.897, n = 14, p = 0.004, ES r = 0.78; Z = 2.696, n = 14, p = 0.007, ES r = 0.72; Z = 1.977, n = 14, p = 0.048, ES r = 0.53, respectively).

**Table 1:** Differences in oxy-Hb level changes between S-VMI and P-VMI Data are expressed as median (IQR); unit, mm; oxyHb, oxy-hemoglobin; Ch6, premotor and supplementary motor cortices; Ch13, supramarginal gyrus; Ch30, superior parietal lobule; S-VMI, standard-visual-motor illusion; P-VMI, power-visual-motor illusion; ES, effect size. ＊Large effect size.

Channel	Period	S-VMI	P-VMI	p-value	ES(r)
Ch6	Online effect	-0.0014 (0.0076)	0.0004 (0.0036)	0.023	0.61＊
	After effect	-0.0006 (0.0066)	0.0012 (0.0065)	0.004	0.78＊
Ch13	After effect	-0.0017 (0.0098)	0.0008 (0.0040)	0.007	0.72＊
Ch30	After effect	-0.0008 (0.013)	0.00256 (0.00172)	0.048	0.53＊

Kinesthetic illusion and sense of embodiment

**Figure 4:**
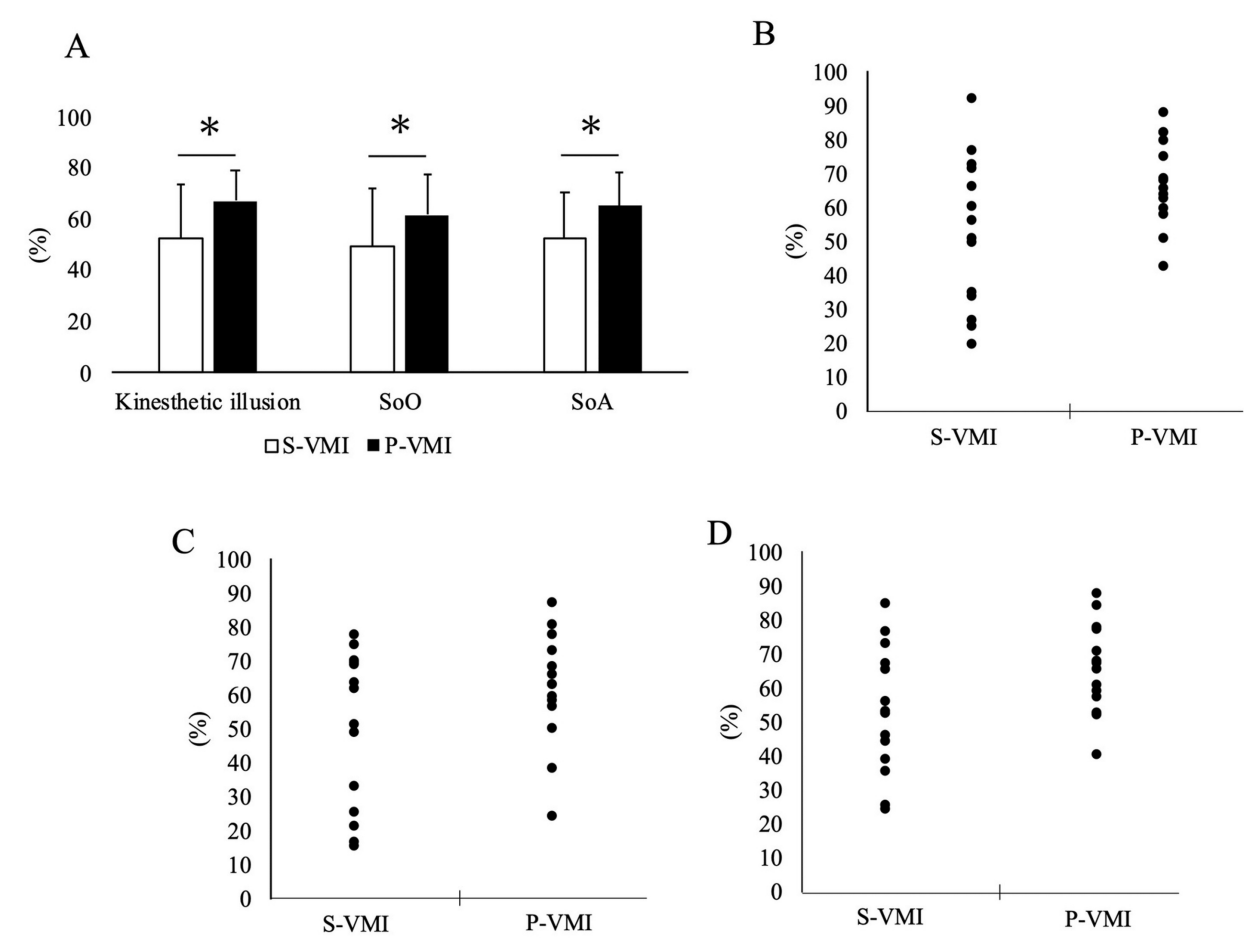
VAS value of Kinesthetic illusion and sense of embodiment. A: Kinesthetic illusion, SoO, and SoA bar graphs (mean ± SD) are shown. In all cases, P-VMI was significantly higher than S-VMI. B: Scatter plots of S-VMI and P-VMI in Kinesthetic illusion. C: Scatter plots of S-VMI and P-VMI in SoO. D: Scatter plots of S-VMI and P-VMI in SoA. S-VMI: standard-visual-motor illusion, P-VMI: power-visual-motor illusion, SoO: sense of ownership, SoA: sense of agency, VAS: visual analog scale.

The results are shown in Figure 4. P-VMI had significantly higher degrees of kinesthetic illusion, SoO, and SoA compared to S-VMI. (Kinesthetic illusion: S-VMI vs. P-VMI t(13) = -2.7, p = 0.018, S-VMI 52.5 ± 22.1 mm, P-VMI 67.4 ± 12.6 mm; SoO: S-VMI vs. P-VMI t(13) = -2.2, p = 0.040, S-VMI 50.0 ± 22.9 mm, P-VMI 61.9 ± 16.6 mm; SoA: S-VMI vs. P-VMI t(13) = -2.8, p = 0.014, S-VMI 53.1 ± 18.6 mm, P-VMI 65.6 ± 13.2 mm).

Relationship between brain activity and kinesthetic illusion and sense of embodiment

**Figure 5:**
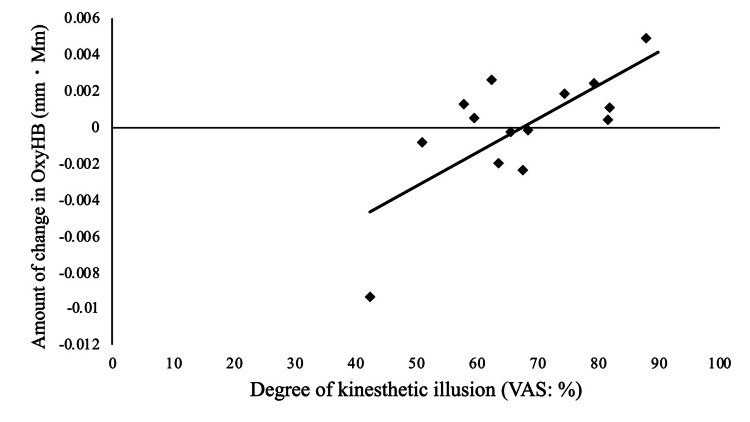
Relationship between brain activity and kinesthetic illusion and sense of embodiment. In the after-effect of P-VMI, a significant correlation was reported between the degree of kinesthetic illusion and activity in the superior parietal lobule (Ch30) (r = 0.604, p = 0.02). VAS: visual analog scale.

A significant positive correlation was found between the activity of the right superior parietal lobule (Ch 30) and the degree of kinesthetic illusion in the after-effect of P-VMI (p = 0.020, r = 0.604; Figure 5). No other significant correlations were observed.

## Discussion

This study confirmed that changes in brain activity occurred due to variations in the intensity of the presented joint movements in VMI. P-VMI resulted in greater activity in the left premotor area, supplementary motor area, supramarginal gyrus, and right superior parietal lobule compared to S-VMI. These results indicate that increasing the motor intensity of the presented joint movements may enhance activity in motor-related areas.

In a previous study of mirror therapy using virtual reality, activation of the primary motor somatosensory cortex and supplementary motor cortex ipsilateral to the limb producing the illusion was observed, similar to the results of this study [1]. The VMI intervention involving the left ankle joint in our study resulted in increased activity in the left (ipsilateral) premotor and supplementary motor cortices for P-VMI over S-VMI. Previous studies have shown that enhancing exercise intensity during toe flexion in healthy participants increases activity in motor-associated areas [10]. High-intensity exercise requires greater activation of functional areas of the cortex to recruit more motor neurons for muscle contraction and power regulation [19]. Thus, motor-related cortical regions, including the sensorimotor cortex, premotor cortex, and supplementary motor area, are highly activated during resistance training as major cortical regions inducing motor control. This increased activation is also thought to occur because strong muscle contractions increase activity in both proximal and distal muscles [20]. Proximal muscles are associated with the cortical reticular pathway (medial motor system), projecting from the premotor and supplementary motor areas ipsilateral to the limb where movement is induced, directing them to bilateral trunk muscles [21]. Given that P-VMI presented images of increased joint motion intensity with resistance applied to the ankle joint, it might have stimulated activity in the ipsilateral premotor and supplementary motor areas associated with the proximal muscles. In fact, while S-VMI showed no effect on trunk movement for hemiplegic patients, P-VMI enhanced the angular velocity of trunk forward tilt during the sit-to-stand process [11]. Therefore, VMI with augmented joint movement intensity may activate brain regions associated with both proximal and distal muscles, potentially contributing to improved trunk function.

P-VMI also resulted in higher activity in the left supramarginal gyrus compared to S-VMI. The supramarginal gyrus is part of the inferior parietal lobule and a constituent of the Mirror Neuron System (MNS) [22]. Action observation therapy (AOT) has been reported as an intervention method that uses the MNS as its neural basis, similar to VMI, as both tasks involve movement observation in a video image and stimulate motor imagery from visual stimuli. In terms of activated brain regions, VMI activates the fronto-parietal network [4], a region overlapping with the MNS. A previous study showed greater activity in the inferior parietal lobule, the core of the MNS, when observing a video of a strongly gripped hand compared to a lightly gripped one [22]. Similarly, P-VMI might have increased the activity of the supramarginal gyrus, part of the MNS, in this study.

P-VMI significantly increased the activity level in the right (contralateral) superior parietal lobule compared with S-VMI and also significantly increased the perception of somatization sensation (SoO and SoA). Ohata et al. reported that the right inferior parietal lobule plays a critical role in SoA [23]. This study showed increased activity in the right superior parietal lobule, a region previously reported to have connectivity with the inferior parietal lobule [24]. Therefore, this study may confirm the activity of the inferior parietal lobule and the connectivity of the superior parietal lobule. Furthermore, prior research has revealed that imparting a sense of effort tends to enhance SoA [25]. As P-VMI displayed images of an effortful ankle dorsiflexion movement with resistance, the SoA might have been intensified more than S-VMI. Regarding SoO, Shimada et al. reported that the superior parietal lobule activates when vision and proprioception temporally synchronize, indicating its importance in generating SoO [26]. Furthermore, it has been reported that SoO and SoA interact in the insula, a central brain region responsible for the overall perception of the body and behavior [27]. Therefore, although this study was unable to assess insular cortical activity with fNIRS, the interaction between SoO and SoA might have contributed to their mutual enhancement. The superior parietal lobule has also been reported to be involved in visual imagery and mental transformation of the body [28]. VMI has a visual imagery component because the joint movements in the image induce the image, and P-VMI has a high SoO, which may have led to the mental transformation of the body because the feet in the image felt like one’s own feet.

Compared to S-VMI, P-VMI exhibited increased activity in the right (contralateral) superior parietal lobule in the After-effect, and this region showed a significant positive correlation with the degree of kinesthetic illusion. Naito et al. also reported a significant activation of the right fronto-parietal lobe network when illusion was experienced with either hand [29]. Therefore, this study’s findings align with previous research reporting an association between the right hemisphere and kinesthetic illusions. Moreover, Iwabe et al. examined changes in oxy-Hb in the sensorimotor domain over time during 100% MVC and 50% MVC conditions in a hand-gripping task [30]. They found a significantly faster decay rate in the 50% MVC condition, and that oxy-Hb in the 100% MVC condition peaked just before and after task completion [30]. Since VMI shows brain activity similar to actual exercise [4], it is possible that P-VMI, with its increased joint movement intensity, increased brain activity in the After-effect. Earlier studies have shown that physical or occupational therapy following VMI is effective in improving the functionality of paralyzed upper extremities and ankles [2,3]. Therefore, P-VMI may be beneficial for clinical applications, given that the kinesthetic illusion continues after the intervention.

This study has some limitations. First, the study included a small sample size. Second, fNIRS is unable to provide information about activity in brain regions deeper than the cerebral cortex, restricting its applicability. Therefore, brain activity should be examined using functional magnetic resonance imaging in future studies. Third, our study only involved healthy participants, which may not accurately reflect the brain activity of actual hemiplegic patients. Therefore, we believe that future studies should include hemiplegic patients. Fourth, muscle activity was not measured during the measurement of brain activity by fNIRS, and pure brain activity was not measured. Fifth, we only evaluated changes in brain activity with VMI and did not evaluate changes in motor function. In the future, brain activity and motor function should be evaluated simultaneously to expand the potential of VMI as a rehabilitation tool. Sixth, participants did not assess the effects of performing similar tasks repeatedly on their brain activity or their ability to concentrate during the experiment.

## Conclusions

The comparison of brain activity between S-VMI and P-VMI demonstrated activation of the medial motor system, the MNS, and the brain areas involved in somatization and illusion sensations in P-VMI. Therefore, P-VMI may be a more effective VMI intervention than S-VMI in clinical settings.

## Figures and Tables

**Figure 6:**
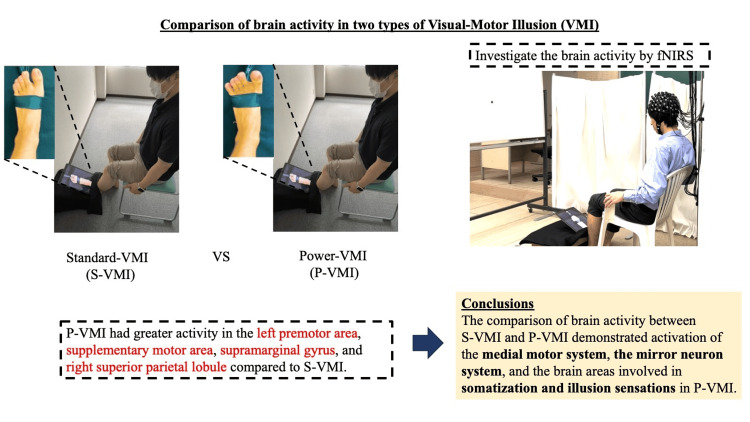
Graphical abstract S-VMI: standard visual-motor illusion, P-VMI: power visual-motor illusion, fNIRS: functional near-infrared spectroscopy.
